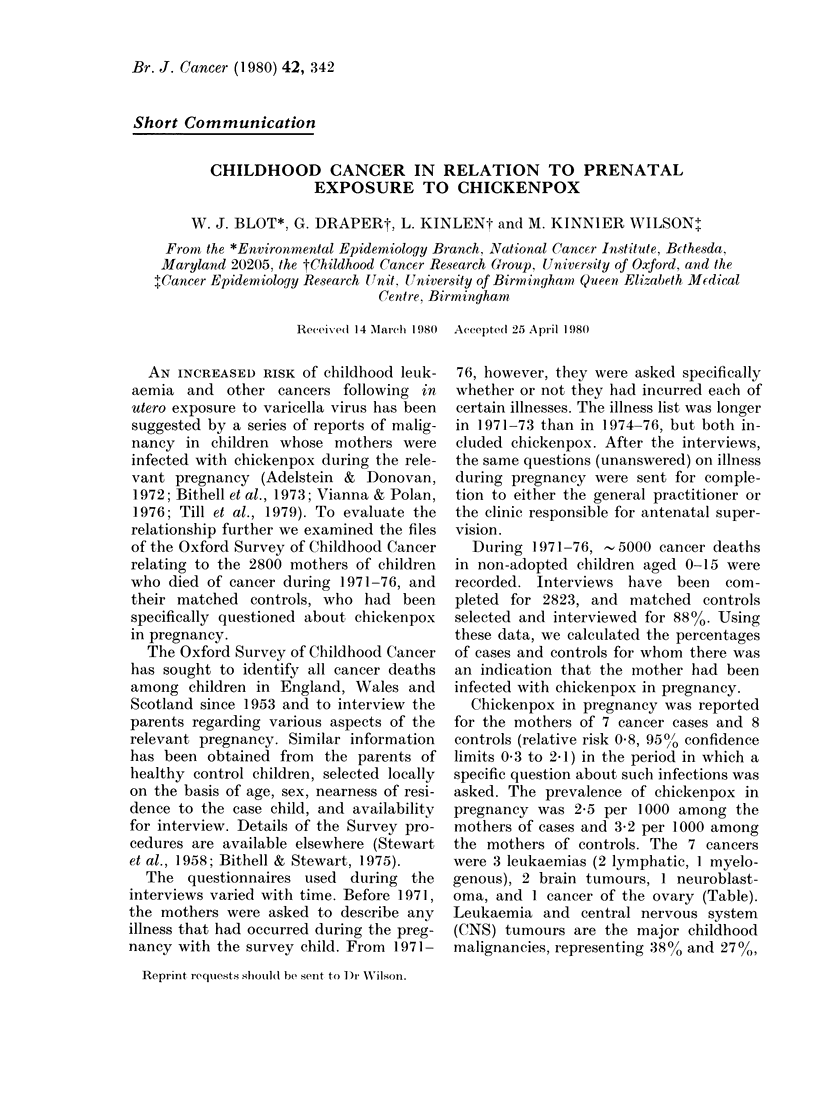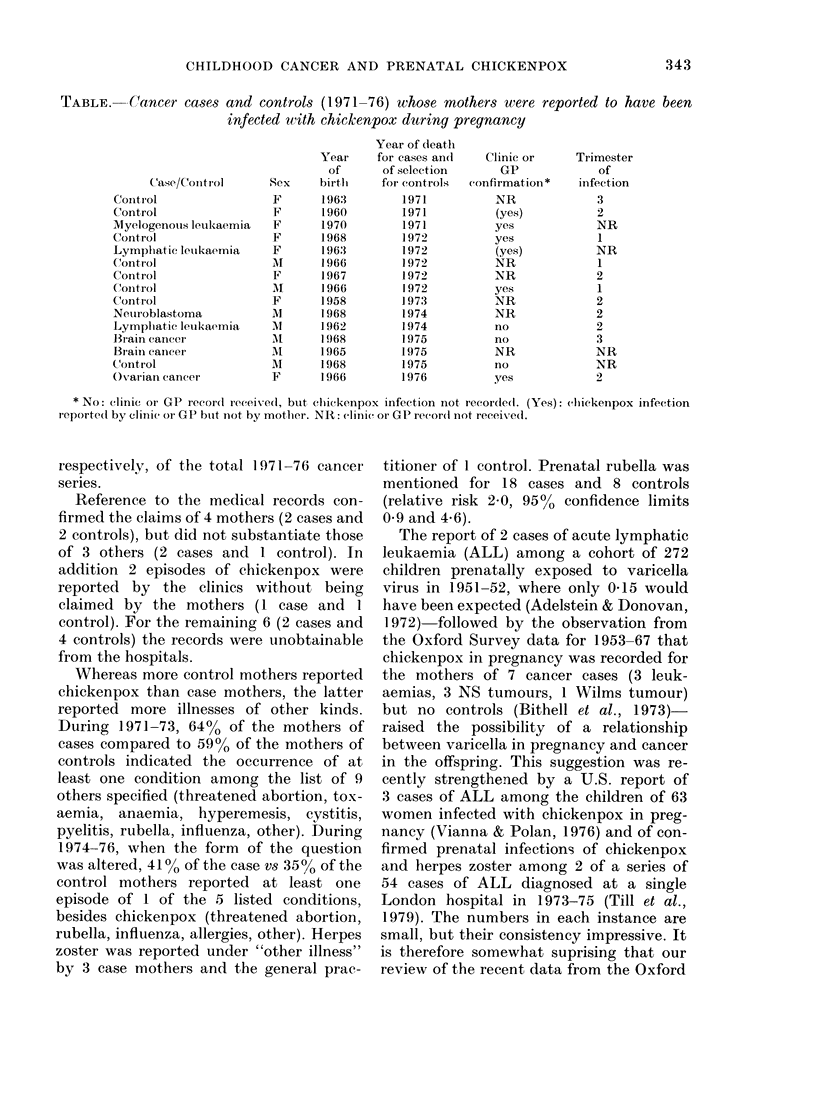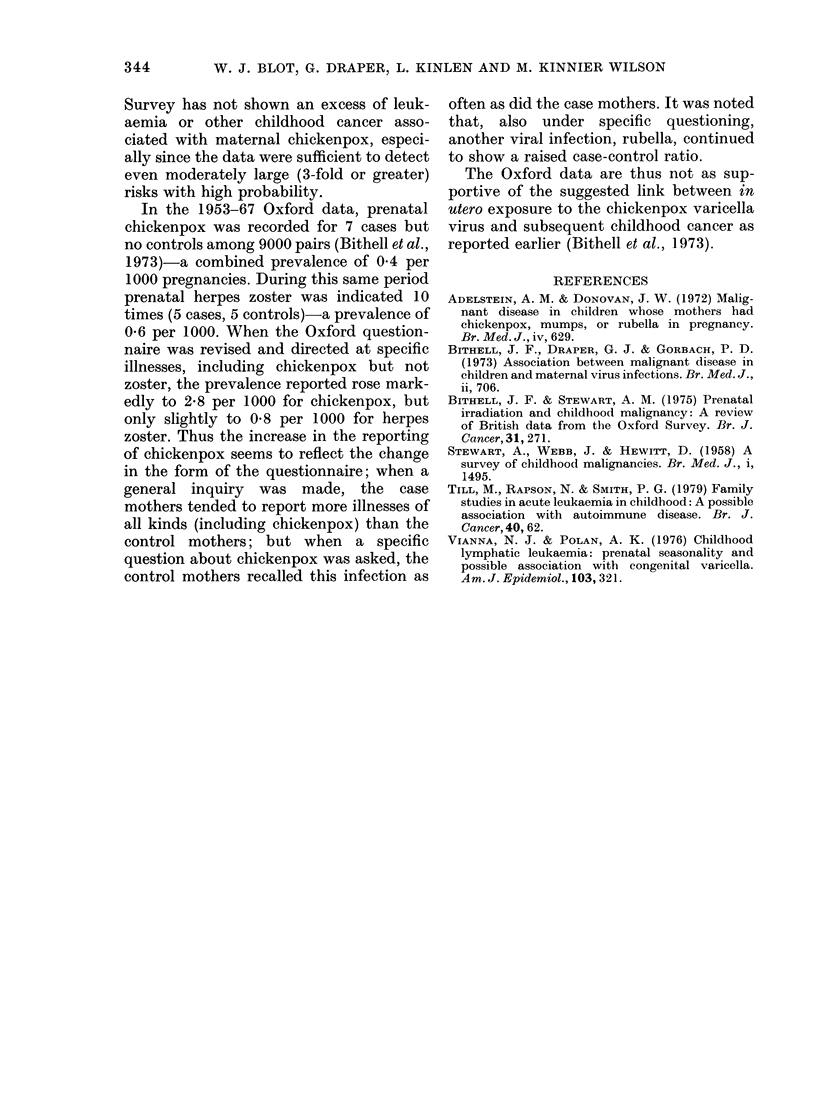# Childhood cancer in relation to prenatal exposure to chickenpox.

**DOI:** 10.1038/bjc.1980.236

**Published:** 1980-08

**Authors:** W. J. Blot, G. Draper, L. Kinlen, M. K. Wilson


*Br. J. Cancer* (1980) **42**, 342

***Short Communication***

### CHILDHOOD CANCER IN RELATION TO PRENATAL EXPOSURE TO CHICKENPOX

W. J. BLOT*, G. DRAPER†, L. KINLEN† and M. KINNIER WILSON‡

*From the *Environmental Epidemiology Branch, National Cancer Institute, Bethesda, Maryland* 20205, *the †Childhood Cancer Research Group, University of Oxford, and the ‡Cancer Epidemiology Research Unit, University of Birmingham Queen Elizabeth Medical Centre, Birmingham*

Received 14 March 1980 Accepted 25 April 1980

An increased risk of childhood leukaemia and other cancers following *in utero* exposure to varicella virus has been suggested by a series of reports of malignancy in children whose mothers were infected with chickenpox during the relevant pregnancy (Adelstein & Donovan, 1972; Bithell *et al.*, 1973; Vianna & Polan, 1976; Till *et al.*, 1979). To evaluate the relationship further we examined the files of the Oxford Survey of Childhood Cancer relating to the 2800 mothers of children who died of cancer during 1971–76, and their matched controls, who had been specifically questioned about chickenpox in pregnancy.

The Oxford Survey of Childhood Cancer has sought to identify all cancer deaths among children in England, Wales and Scotland since 1953 and to interview the parents regarding various aspects of the relevant pregnancy. Similar information has been obtained from the parents of healthy control children, selected locally on the basis of age, sex, nearness of residence to the case child, and availability for interview. Details of the Survey procedures are available elsewhere (Stewart *et al.*, 1958; Bithell & Stewart, 1975).

The questionnaires used during the interviews varied with time. Before 1971, the mothers were asked to describe any illness that had occurred during the pregnancy with the survey child. From 1971–76, however, they were asked specifically whether or not they had incurred each of certain illnesses. The illness list was longer in 1971–73 than in 1974–76, but both included chickenpox. After the interviews, the same questions (unanswered) on illness during pregnancy were sent for completion to either the general practitioner or the clinic responsible for antenatal supervision.

During 1971–76, ~5000 cancer deaths in non-adopted children aged 0–15 were recorded. Interviews have been completed for 2823, and matched controls selected and interviewed for 88%. Using these data, we calculated the percentages of cases and controls for whom there was an indication that the mother had been infected with chickenpox in pregnancy.

Chickenpox in pregnancy was reported for the mothers of 7 cancer cases and 8 controls (relative risk 0·8, 95% confidence limits 0·3 to 2·1) in the period in which a specific question about such infections was asked. The prevalence of chickenpox in pregnancy was 2·5 per 1000 among the mothers of cases and 3·2 per 1000 among the mothers of controls. The 7 cancers were 3 leukaemias (2 lymphatic, 1 myelogenous), 2 brain tumours, 1 neuroblastoma, and 1 cancer of the ovary (Table). Leukaemia and central nervous system (CNS) tumours are the major childhood malignancies, representing 38% and 27%,

Reprint requests should be sent to Dr Wilson.

TABLE.—*Cancer cases and controls* (1971–76) *whose mothers were reported to have been infected with chickenpox during pregnancy*

| Case/Control | Sex | Year of birth | Year of death for cases and of selection for controls | Clinic or GP confirmation* | Trimester of infection |
|---|---|---|---|---|---|
| Control | F | 1963 | 1971 | NR | 3 |
| Control | F | 1960 | 1971 | (yes) | 2 |
| Myelogenous leukaemia | F | 1970 | 1971 | yes | NR |
| Control | F | 1968 | 1972 | yes | 1 |
| Lymphatic leukaemia | F | 1963 | 1972 | (yes) | NR |
| Control | M | 1966 | 1972 | NR | 1 |
| Control | F | 1967 | 1972 | NR | 2 |
| Control | M | 1966 | 1972 | yes | 1 |
| Control | F | 1958 | 1973 | NR | 2 |
| Neuroblastoma | M | 1968 | 1974 | NR | 2 |
| Lymphatic leukaemia | M | 1962 | 1974 | no | 2 |
| Brain cancer | M | 1968 | 1975 | no | 3 |
| Brain cancer | M | 1965 | 1975 | NR | NR |
| Control | M | 1968 | 1975 | no | NR |
| Ovarian cancer | F | 1966 | 1976 | yes | 2 |

* No: clinic or GP record received, but chickenpox infection not recorded. (Yes): chickenpox infection reported by clinic or GP but not by mother. NR: clinic or GP record not received.

respectively, of the total 1971–76 cancer series.

Reference to the medical records confirmed the claims of 4 mothers (2 cases and 2 controls), but did not substantiate those of 3 others (2 cases and 1 control). In addition 2 episodes of chickenpox were reported by the clinics without being claimed by the mothers (1 case and 1 control). For the remaining 6 (2 cases and 4 controls) the records were unobtainable from the hospitals.

Whereas more control mothers reported chickenpox than case mothers, the latter reported more illnesses of other kinds. During 1971–73, 64% of the mothers of cases compared to 59% of the mothers of controls indicated the occurrence of at least one condition among the list of 9 others specified (threatened abortion, toxaemia, anaemia, hyperemesis, cystitis, pyelitis, rubella, influenza, other). During 1974–76, when the form of the question was altered, 41% of the case *vs* 35% of the control mothers reported at least one episode of 1 of the 5 listed conditions, besides chickenpox (threatened abortion, rubella, influenza, allergies, other). Herpes zoster was reported under "other illness" by 3 case mothers and the general practitioner of 1 control. Prenatal rubella was mentioned for 18 cases and 8 controls (relative risk 2·0, 95% confidence limits 0·9 and 4·6).

The report of 2 cases of acute lymphatic leukaemia (ALL) among a cohort of 272 children prenatally exposed to varicella virus in 1951–52, where only 0·15 would have been expected (Adelstein & Donovan, 1972)—followed by the observation from the Oxford Survey data for 1953–67 that chickenpox in pregnancy was recorded for the mothers of 7 cancer cases (3 leukaemias, 3 NS tumours, 1 Wilms tumour) but no controls (Bithell *et al.*, 1973)—raised the possibility of a relationship between varicella in pregnancy and cancer in the offspring. This suggestion was recently strengthened by a U.S. report of 3 cases of ALL among the children of 63 women infected with chickenpox in pregnancy (Vianna & Polan, 1976) and of confirmed prenatal infections of chickenpox and herpes zoster among 2 of a series of 54 cases of ALL diagnosed at a single London hospital in 1973–75 (Till *et al.*, 1979). The numbers in each instance are small, but their consistency impressive. It is therefore somewhat suprising that our review of the recent data from the Oxford

Survey has not shown an excess of leukaemia or other childhood cancer associated with maternal chickenpox, especially since the data were sufficient to detect even moderately large (3-fold or greater) risks with high probability.

In the 1953–67 Oxford data, prenatal chickenpox was recorded for 7 cases but no controls among 9000 pairs (Bithell *et al.*, 1973)—a combined prevalence of 0·4 per 1000 pregnancies. During this same period prenatal herpes zoster was indicated 10 times (5 cases, 5 controls)—a prevalence of 0·6 per 1000. When the Oxford questionnaire was revised and directed at specific illnesses, including chickenpox but not zoster, the prevalence reported rose markedly to 2·8 per 1000 for chickenpox, but only slightly to 0·8 per 1000 for herpes zoster. Thus the increase in the reporting of chickenpox seems to reflect the change in the form of the questionnaire; when a general inquiry was made, the case mothers tended to report more illnesses of all kinds (including chickenpox) than the control mothers; but when a specific question about chickenpox was asked, the control mothers recalled this infection as often as did the case mothers. It was noted that, also under specific questioning, another viral infection, rubella, continued to show a raised case-control ratio.

The Oxford data are thus not as supportive of the suggested link between *in utero* exposure to the chickenpox varicella virus and subsequent childhood cancer as reported earlier (Bithell *et al.*, 1973).

### REFERENCES

ADELSTEIN, A. M. & DONOVAN, J. W. (1972) Malignant disease in children whose mothers had chickenpox, mumps, or rubella in pregnancy. *Br. Med. J.*, iv, 629.

BITHELL, J. F., DRAPER, G. J. & GORBACH, P. D. (1973) Association between malignant disease in children and maternal virus infections. *Br. Med. J.*, ii, 706.

BITHELL, J. F. & STEWART, A. M. (1975) Prenatal irradiation and childhood malignancy: A review of British data from the Oxford Survey. *Br. J. Cancer*, **31**, 271.

STEWART, A., WEBB, J. & HEWITT, D. (1958) A survey of childhood malignancies. *Br. Med. J.*, i, 1495.

TILL, M., RAPSON, N. & SMITH, P. G. (1979) Family studies in acute leukaemia in childhood: A possible association with autoimmune disease. *Br. J. Cancer*, **40**, 62.

VIANNA, N. J. & POLAN, A. K. (1976) Childhood lymphatic leukaemia: prenatal seasonality and possible association with congenital varicella. *Am. J. Epidemiol.*, **103**, 321.